# Otago Exercise Program as an Adjunct to Routine Physiotherapy in a Patient With Tibial Plateau Injury: A Case Report

**DOI:** 10.7759/cureus.44136

**Published:** 2023-08-25

**Authors:** Neha Arya, Pallavi Harjpal

**Affiliations:** 1 Physiotherapy, Ravi Nair Physiotherapy College, Datta Meghe Institute of Higher Education and Research, Wardha, IND; 2 Neurophysiotherapy, Ravi Nair Physiotherapy College, Datta Meghe Institute of Higher Education and Research, Wardha, IND

**Keywords:** rehabilitation, otago exercise, strengthening, physiotherapy, tibial plateau

## Abstract

The tibia is a long bone in the lower limb. Tibial fractures account for approximately 20% of adults and 8% of older people. A tibial plateau fracture may result from low-energy forces, most commonly in older people with low bone density. The tibial plateau fractures vary widely, from stable non-displaced fractures with little soft tissue damage to severely comminated unstable fractures with severe soft tissue damage. Fractures of the tibia plateau had a significant impact on patients' lives, lowering their quality of life and limiting their participation in sports. Other effects of the injury itself, such as arthritis later developing, muscle, bone wasting, and joint stiffness, can have an impact on patients' lives. For these patients, physical therapy can target areas to improve some of such conditions. A 50-year-old female was diagnosed with lateral tibial plateau injury in the left knee and post tibial plateau injury in the right knee joint on an X-ray after a road accident, and a bilateral above-knee cast was applied for four weeks, following a period of non-weight-bearing. Along with this, a physiotherapy treatment plan was advised, which included a variety of exercises, electrotherapy, and an Otago exercise program, which resulted in pain reduction and improvements in range of motion (ROM), strength, balance, and gait ability. A structured physiotherapy program with an Otago exercise program gradually improved the functional goals, balance, and gait patterns progressively.

## Introduction

The tibia is the second longest bone of the lower limb [1]. The tibial plateau of the knee joint is one of the most important weight-bearing structures. These fractures are proximal tibia intra-articular knee fractures [2]. Damage can be due to direct impact injury or indirect mechanisms such as axial loading. The most common causes are traffic accidents and sports injuries, which result in high-energy trauma and direct injury to surrounding soft tissue and fractures [3]. The front surface of the lateral plateau, which is seen when the knee is extended, makes it more prone to injury [2]. A tibial plateau fracture may result from low-energy forces, most frequently in elderly patients with poor bone quality. A depressed type of plateau fracture commonly results from this injury, which is most frequently seen in women over 50 with osteoporosis [3].

These patients can be divided into two main age groups. First are patients who are young males, who suffer injuries following high-energy trauma, and old women who suffer these injuries following low-energy injuries. The primary contributing factor in the first group of patients' injuries is high-energy trauma that increases torsional forces on the proximal tibia. Although minimal forces are applied during trauma, osteoporosis-related bone fragility is the primary contributing factor for the older patient population [4].

Physiotherapy is an important component of patients' rehabilitation as it will help them to return to pre-injury activity. This can help prevent some of these issues or focus on the areas where these patients require assistance and good outcomes. The patients' quality of life and ability to participate in sports were significantly affected by tibia plateau fractures, which also decreased their quality of life. Early mobilization and range-of-motion training for the knee joint is crucial, and this has been demonstrated in the literature for a long time [4].

Severe knee stiffness is a recognized complication of injury around the knee. The aim of treatment for a stiff knee is to get it moving normally again without doing more harm to the joint or nearby structures [5]. This case report describes a lateral tibial plateau injury and posterior plateau fracture caused by a road traffic accident in a 50-year-old female who was managed conservatively with a cast and then underwent physiotherapy rehabilitation using the proper rehabilitation protocol.

## Case presentation

Patient information

A 50-year-old female with left-hand dominance met with an accident with a direct fall upon her knees in December 2022. The patient took rest at home as there were no external injuries but pain and swelling gradually increased in both knees. After consultation with an orthopedic surgeon on the next day, an X-ray was done, which revealed a lateral tibial plateau injury on the right side and a posterior column tibial plateau fracture on the left side. The patient underwent conservative management with a bilateral long knee cast for six weeks. Physiotherapy intervention was started on the third-day post-episode. The cast was removed after one month of the episode, and an X-ray was taken. The patient was advised for non-weight bearing for two weeks after the removal of the cast. The patient complained of pain in both knees, describing it as dull-aching and intensifying to a 6/10 when at rest to an 8/10 during movement on the numerical pain rating scale and swelling on both knees with limited mobility. Physiotherapy rehabilitation was continued.

Clinical findings (after case removal)

Informed consent was taken from the patient before the treatment. She was assessed in supine lying. On inspection, both the patient’s legs were extended and slightly abducted, externally rotated and plantar flexed. Visible swelling was present near the knee joint. On palpation, local skin temperature was raised, and Grade 1 tenderness was present as per the grading scale for tenderness.

Initial examination findings

**Figure 1:**
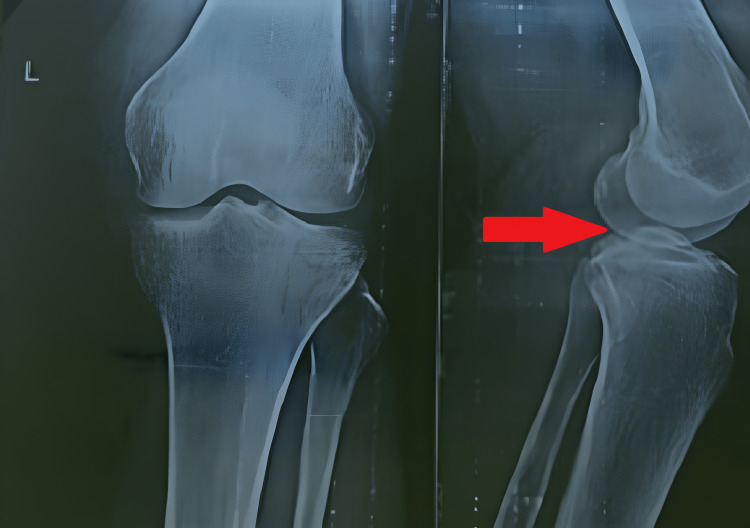
Left knee X-ray revealing injury to the posterior tibial plateau (red arrow)

**Figure 2:**
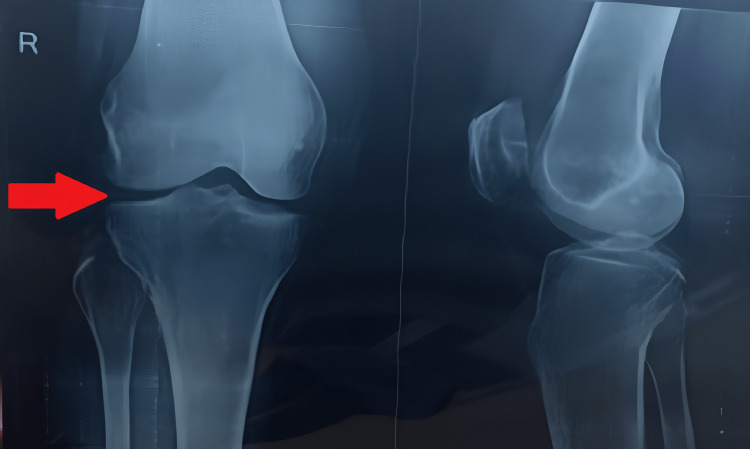
Right knee X-ray revealing injury to the lateral tibial plateau (red arrow)

Range of motion (ROM) and manual muscle testing examination were performed after the removal of the cast. There was a significant decrease in knee joint ROM along with stiffness in both knees, and strength for both quadriceps and hamstrings was also reduced significantly. Berg’s balance scale and dynamic gait index were used to assess balance impairment and gait, and the patient was unable to bear weight on both extremities. The X-ray revealed an injury to the posterior tibial plateau on the left knee (Figure 1) and an injury to the lateral tibial plateau on the right knee (Figure 2).

Physiotherapy

**Figure 3:**
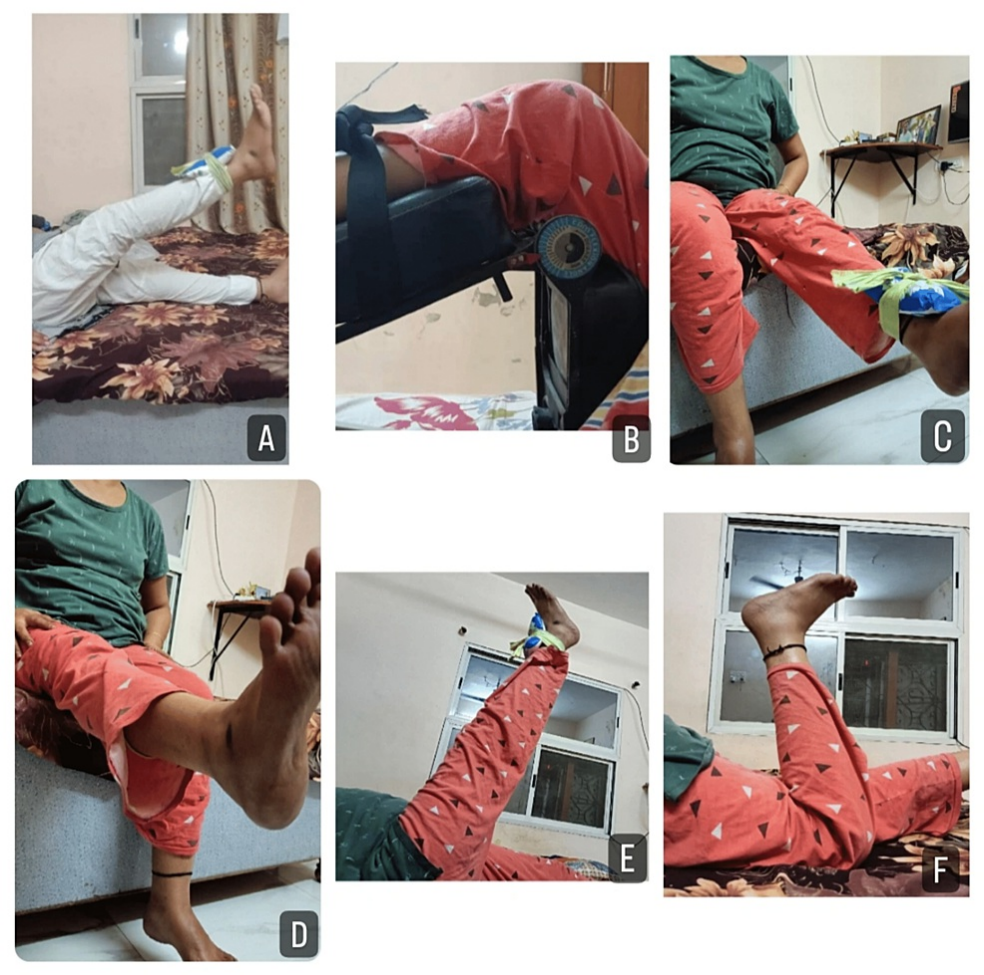
Strengthening and ROM exercises A: hip flexor strengthening with half kg weight cuff, B: continuous passive movement, C: knee extensor strengthening, D: dynamic quads, E: straight leg raises, F: active knee flexion

**Figure 4:**
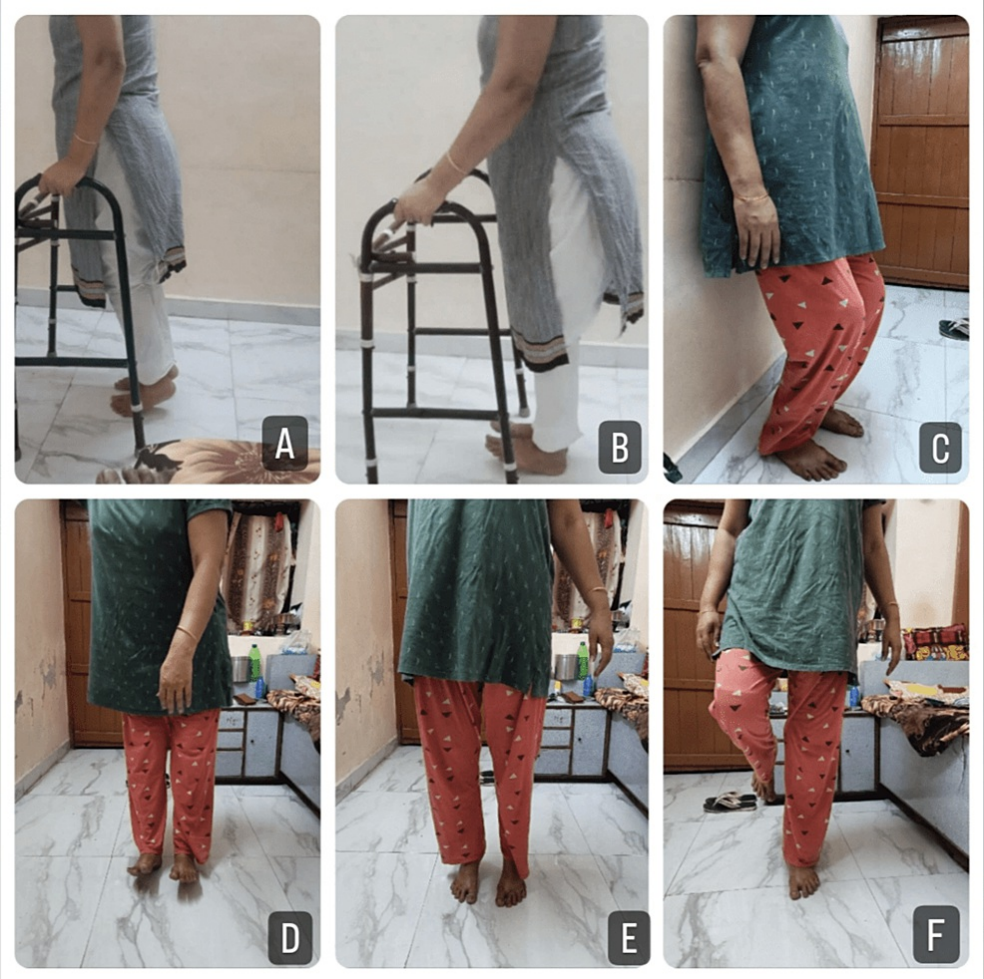
Otago exercise program A: heel raises with the help of a walker, B: toe raises with the help of a walker, C: semi-squats, D: heel raises without assistance, E: toe raises without assistance, F: one-leg standing

A customized rehabilitation plan was created for the patient. The patient received physical therapy rehabilitation for 12 weeks, six days per week. The protocol to be followed during the timeline of the rehabilitation is as follows: from weeks 1-4 during plaster cast (Table 1), from weeks 5-6 during non-weight bearing (Table 2), and from weeks 7-12 during full weight bearing (Tables 3-4). The strengthening exercises and Otago exercise program are provided in Figures 3-4, respectively.

**Table 1:** Weeks 1-4 (during plaster cast) ROM: range of motion

Goals	Strategies	Repetitions
To maintain hip joint mobility, to prevent deep vein thrombosis	ROM exercises, hip abduction (supine lying position), hip extension (side-lying position), ankle toe movements	10 reps x 2 sets (2 times/day)
To maintain the strength of the glutei quadriceps and hamstring muscle	Isometric exercises, static glutes, static quadriceps, static hamstrings	10 reps x 1 set (2 times/day)

**Table 2:** Weeks 5-6 (during non-weight bearing) ROM: range of motion, CPM: continuous passive motion, SLR: straight leg raise, VMO: vastus medialis oblique

Goals	Strategies	Repetitions
To reduce pain and swelling	Cryotherapy	10 mins (2 times/day)
To restore initial ROM for knee joint	CPM for knee (Figure 3; Week 5)	15-20 mins (2 times/day)
To improve patellofemoral mobility	Patellar mobilization	
To increase ROM of knee joint, to increase strength for knee and hip muscles	ROM and strengthening exercises (Week 5): (1) Actively assisted hip-knee flexion, (2) actively assisted knee extension, (3) SLR (Figure 4), (4) hip abduction (side-lying), (5) Hip extension (side-lying)	5 reps x 2 sets (2 times/day) for the whole intervention
To improve the strength of knee extensors, to improve the strength of the knee flexors, to improve the strength of hip joint musculature	Strengthening exercises (Week 6): dynamic quads, Hamstring curls, VMO strengthening, straight leg raises, hip abduction (side-lying), hip extension (prone lying)	10 reps with 5 sec hold x 1 set 10 reps x 2 sets 10 reps x 2 sets 10 reps x 1 set 10 reps x 1 set 10 reps x 1 set

**Table 3:** TABLE 3: Week 7-12 (full weight bearing)

Exercise type	Weeks 7-8	Weeks 9-10
Knee bending	10 repetitions with support 2 sets	10 repetitions without support x 2 sets
Sideways walking	10 steps x 4 sets	
Walking and turning around	Walk and turn around twice (figure of eight)	
Heel walking (Figure 4)	10 steps 2 reps with support	10 steps 2 reps without support
Toe walk (Figure 4)	10 steps 2 reps with support	10 steps 2 reps without support
Sit to stand	10 stands and 2 hands for support	10 reps without support
Tandem stance (heel-toe touch standing)	10 seconds x 2 sets with support	10 seconds x 2 sets with support
Backward walking	10 steps 4 times with support	10 repetitions 4 times without support
Tandem walk (heel-toe walk)	Walk 10 steps 4 times hold support	Walk 10 steps 4 times
One leg standing (Figure 5)	10 seconds with support	10 seconds without support

**Table 4:** Progressive strengthening program after Week 7 VMO: vastus medialis oblique

Progressive strengthening program		
Goals	Strategies	Repetitions
To improve the strength of the quadriceps, hamstrings, hip abductors, and hip flexors	Knee extension	500 gm weight cuff 10 reps x 1 set
	Knee flexion	
	Hip abduction	
	Hip flexion	
	VMO strengthening	
	Wall-supported squats	10 reps x 1 set
	Step up-step down	

Outcome measures

Tables 5-7 depict the outcome measures and follow-ups taken for the patient

**Table 5:** ROM assessment (pre- and post-rehabilitation)

Joint	Right (pre-rehab)		Right (post-rehab)		Left (pre-rehab)		Left (post-rehab)	
	Active	Passive	Active	Passive	Active	Passive	Active	Passive
Hip flexion	0⁰-70⁰	0⁰-90⁰	0⁰-78⁰	0⁰-90⁰	0⁰-75⁰	0⁰-90⁰	0⁰-80⁰	0⁰-90⁰
Extension	0⁰-15⁰	0⁰-15⁰	0⁰-20⁰	0⁰-26⁰	0⁰-15⁰	0⁰-18⁰	0⁰-18⁰	0⁰-25⁰
Abduction	0⁰-45⁰	0⁰-45⁰	0⁰-45⁰	0⁰-45⁰	0⁰-40⁰	0⁰-45⁰	0⁰-45⁰	0⁰-45⁰
Adduction	40⁰-0⁰	45⁰-0⁰	45⁰-0⁰	45⁰-0⁰	40⁰-20⁰	45⁰-0⁰	45⁰-0⁰	45⁰-0⁰
Knee flexion	0⁰-10⁰	0⁰-10⁰	0⁰-110⁰	0⁰-115⁰	0⁰-12⁰	0⁰-12⁰	0⁰-112⁰	0⁰-120⁰
Extension	10⁰-0⁰	10⁰-0⁰	110⁰-0⁰	115⁰-0⁰	12⁰-0⁰	12⁰-0⁰	112⁰-0⁰	120⁰-0⁰
Ankle planter flexion	0⁰-50⁰	0⁰-50⁰	0⁰-50⁰	0⁰-50⁰	0⁰-50⁰	0⁰-50⁰	0⁰-50⁰	0⁰-50⁰
Dorsiflexion	0⁰-16⁰	0⁰-20⁰	0⁰-20⁰	0⁰-20⁰	0⁰-10⁰	0⁰-20⁰	0⁰-20⁰	0⁰-20⁰

**Table 6:** Manual muscle testing (strength) assessment (pre- and post-rehabilitation)

Joint		Left (pre-rehab)	Left (post-rehab)	Right (pre-rehab)	Right (post-rehab)
Hip	Abductors	3	5	3	5
	Adductors	4	5	4	5
	Flexors	4	5	4	5
	Extensors	3-	3-	3-	3-
Knee	Extensors	3-	5	3-	5
	Flexors	3-	4	3-	4
Ankle	Plantar flexors	5	5	5	5
	Dorsi flexors	5	5	5	5

**Table 7:** Outcome measures

Outcome measure	Before rehabilitation	After rehabilitation
Numerical pain rating scale	7/10	2/10
Berg balance scale	0/56	54/56
Dynamic gait index	0/24	22/24

## Discussion

The tibial plateau fracture is one of the most frequent types of injuries. Due to this injury, there is a substantial likelihood that bone microdamage may develop. A fractured tibia requires more recovery time and total bed rest than other bones, which can lead to decreased productivity and psychological conditions in the patient [6]. One of the most common and devastating outcomes of knee injuries is post-fracture stiffness after the application of an above-knee cast [7]. Deficits in proprioception may contribute to the continued risk of falling and disability after a knee fracture. Physical therapy has been shown to enhance proprioceptive sense in patients recovering from orthopedic lower body injuries or surgery. If proprioception in the injured side is impaired following a knee fracture, this may make it easier to focus on particular interventions for fall prevention in the future due to balance issues. [8]. Otago Medical School developed the Otago exercise program, which consists of balance and strength exercises. The Otago exercise program enhances balance and stability in the geriatric population [9].

The patient in the current report underwent structured physical therapy rehabilitation with a variety of exercises from a qualified orthopedic physiotherapist. Cryotherapy and pain medications cause a gradual decrease in pain, enabling the patient to exert more effort during rehabilitation, which gradually enhances muscular strength, ROM, and functional outcomes. The goal of physical therapy was to avoid muscle wasting in both lower limbs while they were immobilized in a long knee cast. After the cast was removed and during the non-weight bearing phase, a continuous passive motion device was used to restore the knee joint's primary ROM. A progressive strengthening program targeting both knee and hip musculature was planned for both extremities [10]. Along with the traditional strengthening program, the Otago exercise program was introduced during the full-weight bearing phase to improve balance and locomotory function. The goal of the above case study was to highlight the significance of essential physiotherapy rehabilitation and the Otago exercise program in achieving functional objectives for the patient and its long-term outlook.

## Conclusions

Normally, tibial plateau fractures are treated surgically; however, due to the minimal findings and injury, this case was treated conservatively with the use of a cast. After 10 weeks of the Otago exercise program combined with physiotherapy, the patient showed significant improvements in her knee function, balance, and gait. She was able to walk independently without pain or assistive devices, and she reported increased confidence and satisfaction with her mobility. She also did not experience any adverse events or complications during the intervention. The Otago exercise program was well-tolerated and accepted by the patient. Thus, it can be concluded that the Otago exercise program is a safe, effective, and feasible adjunct to routine physiotherapy in patients with tibial plateau injury. The Otago exercise program may help accelerate the recovery process, prevent further falls, and improve the quality of life of older adults with lower limb fractures.
